# Oolonghomobisflavans exert neuroprotective activities in cultured neuronal cells and anti-aging effects in *Caenorhabditis elegans*

**DOI:** 10.3389/fnagi.2022.967316

**Published:** 2022-09-07

**Authors:** Shaoxiong Zhang, Chatrawee Duangjan, Tewin Tencomnao, Liangyu Wu, Michael Wink, Jinke Lin

**Affiliations:** ^1^College of Horticulture, Fujian Agriculture and Forestry University, Fuzhou, China; ^2^Natural Products for Neuroprotection and Anti-Ageing Research Unit, Department of Clinical Chemistry, Faculty of Allied Health Sciences, Chulalongkorn University, Bangkok, Thailand; ^3^Institute of Pharmacy and Molecular Biotechnology, Heidelberg University, Heidelberg, Germany; ^4^Leonard Davis School of Gerontology, University of Southern California, Los Angeles, Los Angeles, CA, United States; ^5^Anxi College of Tea Science, Fujian Agriculture and Forestry University, Fuzhou, China

**Keywords:** oolonghomobisflavans, oxidative stress, antioxidants, anti-aging, neuroprotective, neurite outgrowth

## Abstract

Potential health benefits of tea has attracted significant scientific and public attention worldwide. Tea polyphenols are considered as natural promising complementary therapeutical agents for neurodegenerative diseases. However, the anti-neurodegeneration or anti-aging activities of oolong tea polyphenols have not been investigated. The current study aims to document beneficial effects of oolong tea polyphenols [dimers of epigallocatechin gallate (EGCG), oolonghomobisflavan A (OFA), and oolonghomobisflavan B (OFB)] with neuroprotective and neuritogenesis properties in cultured neuronal (Neuro-2a and HT22) cells and *Caenorhabditis elegans* models. *In vitro*, we found that the compounds (EGCG, OFA, and OFB) protect against glutamate-induced neurotoxicity *via* scavenging radical activity, suppression intracellular ROS and up-regulation of antioxidant enzymes. Moreover, the compounds induce neurite outgrowth *via* up-regulate Ten-4 gene expression. Interestingly, OFA and OFB exert stronger neuroprotective and neurite outgrowth properties than EGCG known as an excellent antioxidant agent in tea. *In vivo*, we found that the compounds protect against *C. elegans* Aβ-induced paralysis, chemotaxis deficiency and α-synuclein aggregation. Moreover, the compounds are capable of extending the lifespan of *C. elegans.* OFA and OFB possess both anti-neurodegeneration and anti-aging activities, supporting its therapeutic potential for the treatment of age-related neurodegenerative diseases which need to be studied in more detail in intervention studies.

## Introduction

As aging populations increase rapidly, millions of people around the world are currently affected by neurodegenerative diseases and the disease treatment is expected to grow rapidly as more people live longer (Pérez-Hernández et al., 2016). The most common of neurodegenerative diseases is Alzheimer’s disease (AD) which is associated with age (Pérez-Hernández et al., 2016).

Glutamate is the main excitatory neurotransmitter in the brain while excessive levels of glutamate can cause neuronal cell death through mitochondrial function impairment and accumulation of reactive oxygen species (ROS) (Tobaben et al., 2011; Rocha et al., 2020). Oxidative stress has been reported as a factor in neurodegenerative diseases as well as AD. The accumulation of ROS during aging is closely related with mitochondrial dysfunction leading to neuro-inflammation and neuronal cell death which plays an important role in neurodegenerative diseases especially AD (Bonaterra et al., 2017).

Neuritogenesis involves the growth, differentiation and regeneration of neurites. The impairment of the neurogenesis process affects neurons cell survival and differentiation leading to various neurodegenerative diseases (Park et al., 2015). Neurite outgrowth is an initial process for functional neuron networks including neuronal differentiation and regeneration (Read and Gorman, 2009). Regulation of neurite outgrowth can promote neuronal regeneration from nerve injury or neurological disorders and plays an valuable role in development of therapies for neurodegenerative diseases (Park et al., 2015; Rangsinth et al., 2021).

In brain, reduction of oxidative stress and induction of neuronal differentiation are the key parameters for neuroprotective effects. Moreover, current drug treatment for neurodegenerative diseases is at the risk of loss of function with many unfavorable side effects (Pérez-Hernández et al., 2016).

**FIGURE 1:**
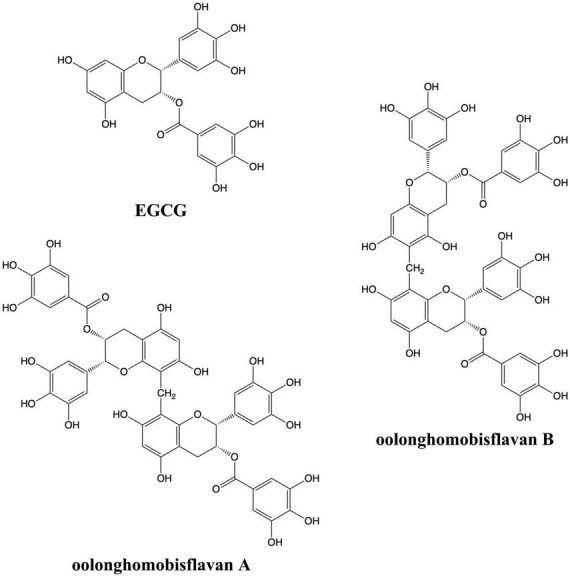
Chemical structures of EGCG, OFA, and OFB.

Plant natural bioactive compounds have turned into an alternative choice to prevent and relieve neurodegenerative diseases. Oolong tea has been reported for many beneficial effects, including anti-hyperglycemic, anti-obesity, anti-bacterial and anti-inflammatory properties (Weerawatanakorn et al., 2015). Oolonghomobisflavan A (OFA) and oolonghomobisflavan B (OFB) have a unique structure as a dimer of epigallocatechin gallate (EGCG) which could be isolated from oolong tea but not from green tea or black tea (Nakai et al., 2005; Figure 1). However, neuroprotective and anti-aging activity of oolong tea polyphenols, OFA and OFB, have not been reported. Anti-aging in this study is concerned with extending the lifespan. This research shows the first proof of longevity promoting effects and neuroprotective properties of OFA and OFB in cultured neuronal cells (Neuro-2a and HT22) and *C. elegans* model.

## Materials and methods

### Chemicals and reagents

OFA (≥98%) and OFB (≥98%) were purchased from Nagara Science (Japan). EGCG (≥95%), Dimethyl sulfoxide (DMSO), Dulbecco’s modified eagle medium (DMEM), Fetal bovine serum (FBS), L-glutamic acid were purchased from Sigma-Aldrich (United States). Agar, tryptone, yeast extract were purchased from Becton, benzaldehyde from Merck, TRIzol^§^ from Invitrogen, and 3-(4,5-Dimethylthiazol-2-yl)-2,5-diphenyltetrazolium bromide (MTT) from Bio Basic.

### Diphenyl-2-picrylhydrazyl assay

The 1,1-diphenyl-2-picrylhydrazyl (DPPH) radical scavenging activity of the EGCG and OFA and OFB were evaluated according to the user’s manual (Peixoto et al., 2016), with a minor modification. The DPPH working solution was prepared in methanol and was mixed with the EGCG and OFA and OFB at a 1:1 ratio by serial methanolic dilution. After 30 min incubation at room temperature under dark condition, the absorbance was measured at 517 nm using an automatic 96 wells plate reader. Radical scavenging activity against DPPH was calculated follow the formula presented below, where A_0_ represent the absorbance of the control, and A_1_ represent the absorbance after adding the extract.


DPPHradicalscavengingeffect(%)=[(A-0A)1/A]0×100.

The results were collected from three independent experiments performed in duplicate and are showed as EC_50_ (μg/ml).

### Cell culture and treatment

Neuro-2a cell line (purchased from The JCRB Cell Bank, Japan) and HT22 cell line (purchased from The Salk Institute, United States) were used as cultured neuronal cell models in this study. Neuro-2a cell line were cultured in DMEM/HamF12 medium contain 1% penicillin-streptomycin solution and 10% FBS. HT22 cell line were cultured in DMEM medium contain 1% penicillin-streptomycin solution and 10% FBS. Neuronal cells were incubated at incubator (37°C, 5% CO_2_).

### Cell viability

MTT and lactate dehydrogenase (LDH) assays were used in investigating cell viability. Cells (1.5 10^4^ cells/well) were seeded in plates (96-well) which contained 100 μl 10% FBS medium overnight and treated with compounds (EGCG, OFA, and OFB) for 1 h.

MTT and LDH assay were done as previously described (Zhang et al., 2020).

### Measurement of neurite outgrowth and neurite-bearing cells

Neuro-2a cells (1.5 10^4^ cells/well) were seeded in 6 wells plate containing 10% FBS and cultured overnight. Then the cell culture medium was removed and cells were washed with PBS. After that, cells were cultured 24 h with compounds under the condition of low nutrition (1% FBS). Photos were taken from around 100 cells/treatment group, the lengths of neurite were measured with Fiji software (NIH, Bethesda, MD, United States). The cells whose neurite length is longer than the diameter of cell body are regard as neurite bearing cells (Park et al., 2015).

### Glutamate induced cell toxicity

The cells were exposed to 1.25–40 mM glutamate for 1–30 h, which decreased cell viability in a time and a dose-dependent manner (Zhang et al., 2020).

Cells were cultured overnight and then treated with or without compounds for 1 h, followed by adding 10 mM glutamate (Neuro-2a cell line) or 5 mM glutamate (HT22 cell line) for 24 or 18 h to induce cell toxicity.

### Quantitative real-time PCR analysis

For neurite outgrowth assay, cells (9 × 10^4^ cells/well) were seeded in 6 wells plates containing 10% FBS medium overnight, and then treated with compounds in 1% FBS for 24 h. For neuroprotective assay, cells (9 × 10^4^ cells/well) were seeded in 6 wells plates containing 10% FBS medium overnight, and then treated with compounds in 10% FBS for 1 h. According to manufacturer’s instructions, total RNA was extracted using TRIzol^®^ Reagent. cDNA was synthesized from 1 μg of total RNA using AccuPower^®^ RT Premix with oligo (dT). All Quantitative real-time PCR (qRT-PCR) reactions were performed in an Exicycler™ 96. PCR settings: 95°C for 15 min, 45–55 cycles of denaturation at 95°C for 15 s, annealing/extension at 55°C for 30 s. The mRNA expression of *GAP-43*, *Ten-4*, *GSTo1*, *GSTa2*, *GPx*, *CAT*, *SOD1*, and *SOD2* (Zhang et al., 2020) were detected by qRT-PCR, β*-actin* was selected for standardization control.

### Measurement of intracellular reactive oxygen species levels in cultured neuronal cells

The protective effect against intracellular ROS accumulation was detected by H2DCFDA staining as previously described (Zhang et al., 2020). Briefly, the cells were seeded in the plates overnight and pre-treated with compounds (EGCG, OFA, and OFB) for 1 h, followed by co-treatment with glutamate for 18 h (Neuro-2a) or 12 h (HT22). The fluorescence intensity was measured under the condition of 485 nm excitation and 535 nm emission. The results are expressed as the percentage of the fluorescence intensity of treatment group compared with negative control group.

### *Caenorhabditis elegans* strains and maintenance

*Caenorhabditis elegans* wild-type (N2), TJ356, TJ375, CL2166 were maintained at 20°C, while CL4176, CL802, CL2355, CL2122 were maintained at 16°C. All the strains were maintained on nematode growth medium (NGM) with *Escherichia coli* OP50 as food source.

### Lifespan assay

Lifespan assay was conducted as previously described (Duangjan et al., 2019b). Age-synchronized L4 larvae were transferred with platinum picker to NGM plates, which contained different concentrations of OFA and OFB and the transfer time was set as day 0. During the egg-laying period, the worms were transferred to a fresh corresponding concentration plate every day to ensure that the offspring produced by worms do not affect the experimental results, after that, the worms were transferred to fresh plate every second day. The numbers of survival and dead worms were recorded every other day during transfer to the fresh plate until all worms died. If there was no response after slightly touching the worm with platinum wire, they were recorded as dead. Worms that produce “internal hatch,” escape from the NGM plate or lost, and were severely damaged during transfer are recorded as “censored.” Each group had at least three replicates, and each replicate consisted of approximately 30 worms. The experiment was conducted in a double-blind manner.

### Measurement of intracellular reactive oxygen species levels in *Caenorhabditis elegans*

Under the condition of 20°C, wild type L1 larvae wild-type (N2) were treated with OFA and OFB (5, 10, 25 μM) for 48 h and exposed to non-lethal concentration of the pro-oxidative juglone (20 μM) for 24 h. After that, 20 μM H_2_DCFDA was added to the worm culture medium and then incubated in the dark for 45 min. Then worms were washed with M9 buffer to remove residual dye. Live images were captured from at least 20 worms per group by fluorescence microscopy. The fluorescence intensities were detected and analyzed (Duangjan et al., 2019b).

### Survival assay under juglone-induced oxidative stress

Survival assay was done as previously described (Abbas and Wink, 2009). Briefly, age-synchronized L1 larvae wild-type (N2) worms were treated with OFA and OFB (5, 10, 25 μM) for 48 h in S-medium. Then, worms were exposed to the pro-oxidant juglone (80 μM) for 24 h, the number of survivors were examined and scored.

### Fluorescence detection and image analysis

The transgenic worms with a target gene promoter fused with a GFP reporter were used in gene expression assay. The fluorescence intensity value emitted by the worm represents the relative expression of the target gene. Worms treated with OFA and OFB need to be cleaned by gently wash with M9 buffer, and then fixed on a glass slide with 100 mM tetramisole hydrochloride. After the cover glass has been placed, a fluorescence microscope (BIOREVO BZ-9000) was used for analysis. Approximately 20 worms were randomly selected for each group and each replicate to be photographed. The average fluorescence intensities value of worms were analyzed by Fiji software.

### The expression of stress-related genes

The transgenic strain TJ375 (HSP-16.2:GFP) and CL2166 (GST-4:GFP) were employed in this assay. Age-synchronized L1 worms were cultured in S-medium containing OFA and OFB (5, 10, 25 μM) for 48 h, followed by exposure to 20 μM juglone for 24 h (Duangjan et al., 2019a).

### Localization of DAF-16

The transgenic strain TJ356 (DAF-16:GFP) was applied to test the localization of DAF-16 in cells (Duangjan et al., 2019a). The synchronized L1 larvae TJ356 were cultured in S-medium and treated with different concentrations of OFA and OFB for 24 h. The worms were collected and photographed under a fluorescence microscope for the analysis of DAF-16 localization, respectively. Data were collected from at least three repetitions, each repetition containing about 20 worms.

### Paralysis assay

Paralysis assay was conducted as previously described (Dostal and Link, 2010). Synchronized eggs of strain CL4176 and its control strain CL802 were maintained at 16°C, on the NGM plates with or without OFA and OFB. The expression of transgene (muscle-specific Aβ_1–42_ in CL4176) was activated by turning up the temperature from 16 to 25°C, begin 48 h after egg laying and continued until paralysis assay finished. The record was executed at 2 h interval until the last worm became paralyzed. The worms that did not move or only moved their head when gently touched with a platinum picker were scored as paralyzed. Time required for half of the nematodes in each group to represent paralysis (PT_50_) was used to quantify the speed of paralysis.

### Chemotaxis assay

The synchronized L1 transgenic *C. elegans* CL2355 and its control strain CL2122 were treated with or without OFA and OFB at 16°C in S-medium with *E. coli* (OP50) for 36 h. The chemotaxis assay was carried out as previously described (Zhang et al., 2020).

### Statistical analysis

All data come from at least three independent experiments. For comparison of two groups, Student’s *t*-test was performed. For comparisons among multiple groups, one-way analysis of variance (ANOVA) followed by Dunnett’s *post-hoc* test (Prism, GraphPad Software, San Diego, CA, United States) were performed. Lifespan assay were performed by Kaplan-Meier in software SPSS24, survival curves were drawn by GraphPad Prism 8 software, and the comparison between the two groups of survival curves was showed by Log Rank (Mantel-Cox) test. *P* < 0.05 is regarded as significant difference between groups.

## Results

### The cytotoxicity of oolonghomobisflavan A and oolonghomobisflavan B in cultured neuronal (HT22 and Neuro-2a) cells

Firstly, to investigate whether EGCG, OFA, and OFB have toxic effects in cultured neuronal (HT22 and Neuro-2a) cells, the cells were treated with the polyphenols and cell viability was analyze by the MTT assay. Different concentrations of EGCG, OFA, and OFB did not cause a significant change in cell viability in Neuro-2a and HT22 cells compared to the negative control group. For subsequent experiments, non-cytotoxic concentrations (5, 10 μM) were chosen (Supplementary Figures 1A,B).

### Oolonghomobisflavan A and oolonghomobisflavan B promotes neurite outgrowth involved in Teneurin-4

**FIGURE 2:**
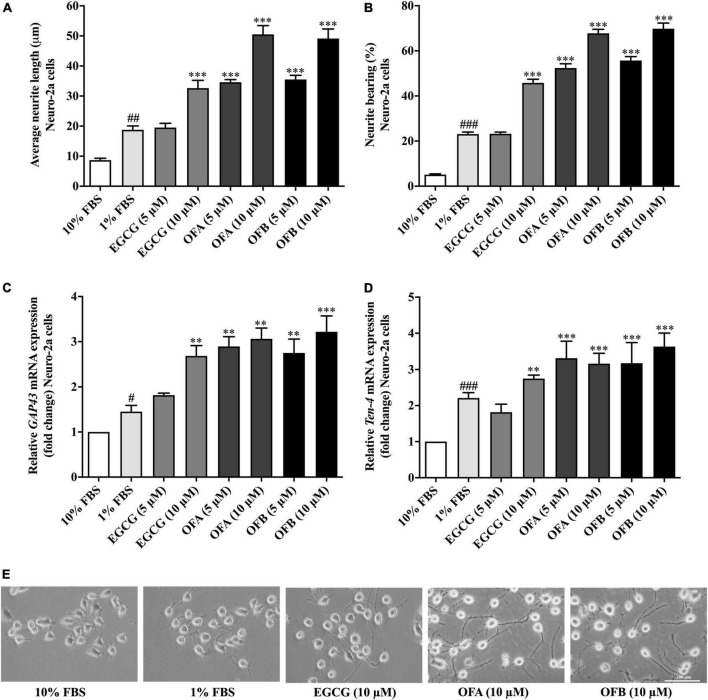
Effect of EGCG, OFA, and OFB on neurite outgrowth. After treated with EGCG, OFA, and OFB in Neuro-2a cells, the average of neurite lengths **(A)** and the percentage of neurite-bearing cells **(B)** were measured. Relative *GAP-43* **(C)** and *Ten-4* **(D)** mRNA expression levels represented as fold change compared to the 10% FBS control in Neuro-2a cells. The images of cell morphology were taken under a 10 magnification microscope **(E)**. β*-Actin* was used in RT-PCR assay as internal control. Results were normalized to 10% FBS control level and shown as the mean ± standard deviation (*n* ≥ 3 independent experiments). ^#^*p* < 0.05, ^##^*p* < 0.01, and ^###^*p* < 0.001 compared to the 10% FBS control; ^**^p < 0.01 and ^***^p < 0.001 compared to the 1% FBS control.

We investigated whether EGCG, OFA, and OFB can induce neurite outgrowth in Neuro-2a cells. The Neuro-2a cells in DMEM supplemented with 10% FBS showed round shape without neurite extension, whereas Neuro-2a cells in DMEM supplemented with 1% FBS (the serum-starved condition) apparently increased the number and the length of neurite (Figures 2A,B).

To investigate the effect of EGCG, OFA, and OFB on neurite outgrowth activity, the cells were maintained in low-serum medium (DMEM supplemented with 1% FBS). The cells treated with EGCG (10 μM), OFA (5, 10 μM), and OFB (5, 10 μM) exhibited significantly increased neurite lengths and neurite bearing cells in a dose-dependent manner, compared to the 1% FBS control group (Figures 2A,B). Interestingly, higher concentrations of OFA (10 μM) and OFB (10 μM) promoted neurite lengths and neurite bearing cells more effectively and were superior to EGCG (10 μM) (*p* < 0.001) (Figures 2A,B,E).

In agreement with neurite outgrowth results, a marker of neurite outgrowth (GAP-43 gene expression) was significantly increased in Neuro-2a cells treated with EGCG (10 μM), OFA (5, 10 μM), and OFB (5, 10 μM), when compared to1% FBS control group (Figure 2C).

To investigate whether Ten-4 expression is involved in EGCG, OFA, and OFB activity to induce neurite growth, the Ten-4 gene expression was examined. The cells treated with EGCG (10 μM), OFA (5, 10 μM), and OFB (5, 10 μM) exhibited significantly increased Ten-4 gene expression, compared to the 1% FBS control group (Figure 2D).

The results suggest that EGCG, OFA, and OFB promote neurite outgrowth, possibly involved the Teneurin-4 transmembrane protein.

### Oolonghomobisflavan A and oolonghomobisflavan B protect against glutamate-induced cytotoxicity

**FIGURE 3:**
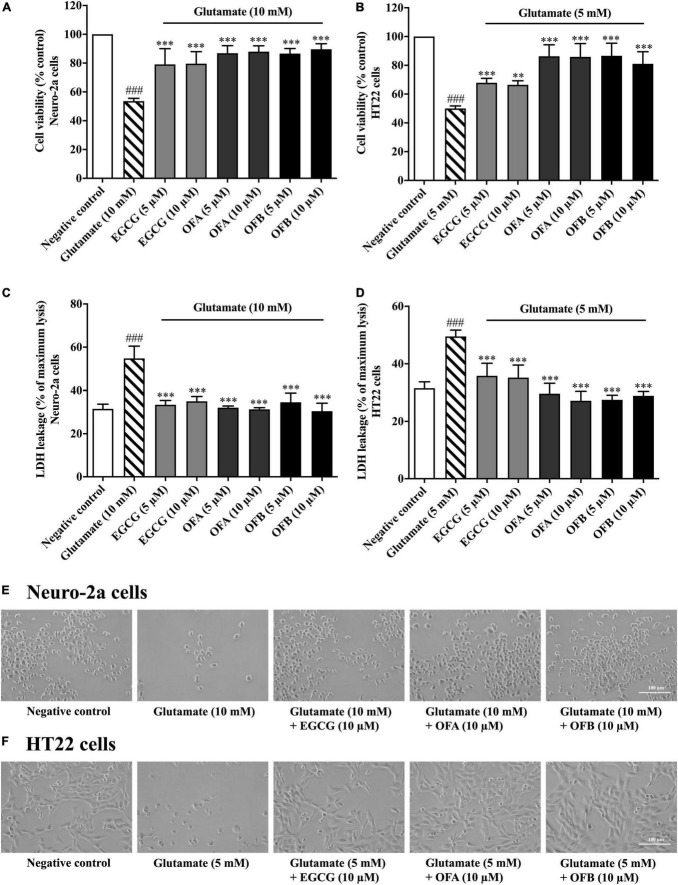
Protective effects of EGCG, OFA, and OFB against glutamate-induced toxicity in Neuro-2a and HT22 cells. Cell viability after treatment with different concentrations of EGCG, OFA, and OFB and exposed to glutamate in Neuro-2a **(A,C)** and HT22 cells **(B,D)**. Samples were exposed to glutamate (5–10 mM) to induce toxicity. Cell morphology was also checked under a 5 magnification microscope **(E,F)**. Results are shown as the mean ± standard deviation (*n* ≥ 3 independent experiments). ^###^*p* < 0.001 compared to the negative control; ^**^p < 0.01 and ^***^p < 0.001, compared to the glutamate treated cells.

To investigate whether EGCG, OFA, and OFB can protect neuronal (Neuro-2a and HT22) cells against glutamate-induced cytotoxicity, the cell viability was determined. The cells treated with varying concentrations of EGCG, OFA, and OFB significantly reduced glutamate-induced cell death, compared to the negative control group (Figures 3A,B). The results were confirmed by the LDH assay (Figures 3C,D) as well as morphological examination (Figures 3E,F). These results suggest that EGCG, OFA, and OFB exert a potent neuroprotective effect against cytotoxicity induced by excessive glutamate in cultured neuronal (Neuro-2a and HT22) cells.

### Oolonghomobisflavan A and oolonghomobisflavan B protect against glutamate-induced oxidative stress

**FIGURE 4:**
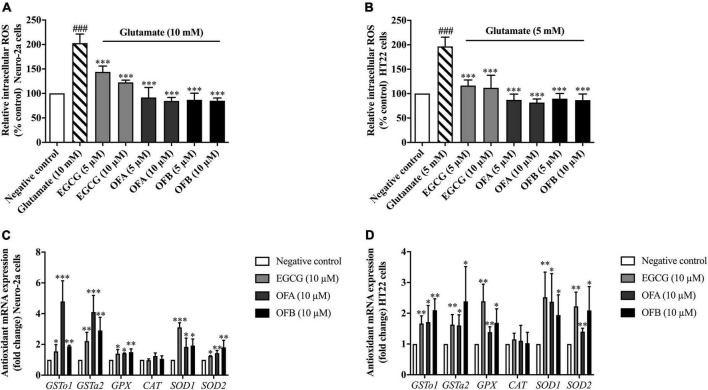
Protective effect of EGCG, OFA, and OFB in Neuro-2a and HT22 cells against glutamate-induced oxidative stress by suppressing intracellular ROS and enhancing the antioxidant gene expression. After treated with EGCG, OFA, and OFB in Neuro-2a **(A)** and HT22 cells **(B)**, the intracellular ROS were measured. Samples were exposed to glutamate (5–10 mM) to induce oxidative stress. The endogenous antioxidant gene expression in Neuro-2a **(C)** and HT22 cells **(D)**. β*-Actin* was used in RT-PCR assay as internal control. Results are shown as the mean ± standard deviation (*n* ≥ 3 independent experiments). ^###^*p* < 0.001 compared to the negative control; *p < 0.05, ^**^p < 0.01, and ^***^p < 0.001 compared to the glutamate treated control.

To investigate whether EGCG, OFA, and OFB could suppress glutamate-induced oxidative stress, their antioxidant properties were explored in Neuro-2a and HT22 cells using intracellular ROS accumulation assay. The cells were exposured to glutamate (10 mM in Neuro-2a cells and 5 mM in HT22 cells) showed significantly elevated intracellular ROS level in Neuro-2a (approximately 2 fold) and HT22 (approximately 1.9 fold) cells, compared to the negative control group (Figures 4A,B). However, the cells treated with all concentration of EGCG, OFA, and OFB significantly reduced the elevated intracellular ROS levels, compared to the glutamate treated group (*P* < 0.001) (Figures 4A,B).

In order to clarify the mechanism of action in antioxidant-mediated neuroprotective effects against glutamate toxicity, the effect of EGCG, OFA, and OFB on the expression of antioxidant as well as phase II enzyme gene were investigated.

Previous results found that EGCG, OFA, and OFB had demonstrated the protective effects against glutamate-induced oxidative stress and toxicity in a concentration-dependent manner with the maximum protective effect at 10 μM (Figures 3A,B, 4A,B). Thus, these concentrations were used to detect expressions of antioxidant genes. The cells treated with EGCG, OFA, and OFB showed significantly up-regulated expressions of antioxidant genes, including *GSTo1*, *GSTa2*, *GPx*, *SOD1*, and *SOD2* (Figures 4C,D). Interestingly, the expression of CAT gene was not significant changed after treated with EGCG, OFA, and OFB in both Neuro-2a and HT22 cells (Figures 4C,D), compared to the negative control.

The results suggest that EGCG, OFA, and OFB protect against glutamate-induced cytotoxicity not only by suppressing intracellular ROS production but also through enhancing the expression of endogenous antioxidant enzymes in cultured neuronal (Neuro-2a and HT22) cells.

### Oolonghomobisflavan A and oolonghomobisflavan B improve the Aβ-induced paralysis in *Caenorhabditis elegans*

**FIGURE 5:**
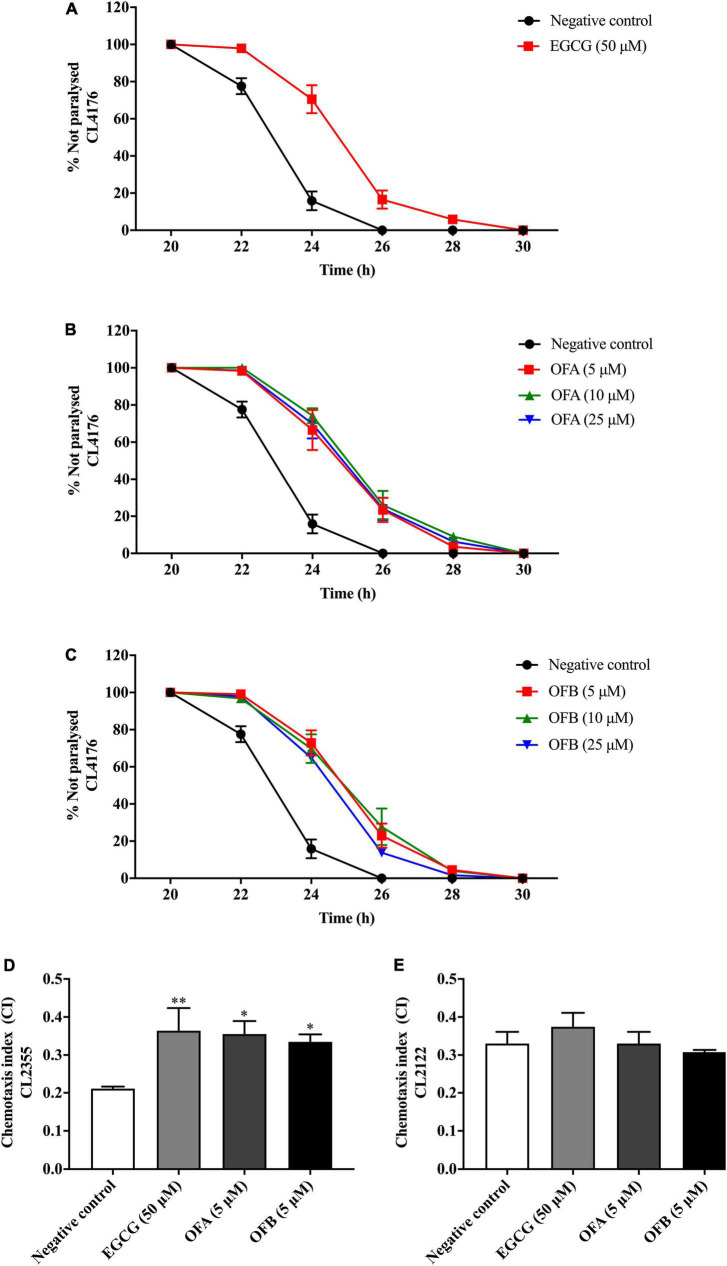
Effect of EGCG, OFA, and OFB on Aβ -induced paralysis in transgenic stain AD model CL4176 **(A–C)**. The indicated times are from the initiation of the temperature shift up. Effect of EGCG, OFA, and OFB on Aβ -induced deficit in chemotaxis behavior in transgenic strain AD model CL2355 **(D)**. EGCG, OFA, and OFB didn’t affect the chemotaxis behaviour of the control strain CL2122 **(E)**. All data are shown as the mean ± SEM (*n* ≥ 3 independent experiments). *p < 0.05, ^**^p < 0.01 compared to the negative control.

We investigated whether EGCG, OFA, and OFB suppress Aβ-induced toxicity in the transgenic *C. elegans* strain CL4176. Worms of CL4176 strain, which express human Aβ_1–42_ peptides, become paralyzed due to the Aβ aggregate accumulation in muscle cells at 25°C, result in oxidative stress (Drake et al., 2003). We found that EGCG, OFA, and OFB extend PT_50_ when compare to negative control. 50 μM EGCG extended PT_50_ by 8.04% (*P* < 0.01), 5 μM OFA by 7.67% (*P* < 0.01), 10 μM OFA by 9.44% (*P* < 0.001), 25 μM OFA by 8.42% (*P* < 0.01), 5 μM OFB by 8.79% (*P* < 0.001), 10 μM OFB by 9.20% (*P* < 0.001), 25 μM OFB by 7.36% (*P* < 0.01) (Figure 5 and Supplementary Table 1). These results suggest that OFA and OFB may contain the potential to protect worms against Aβ-induced toxicity.

### Oolonghomobisflavan A and oolonghomobisflavan B improved the Aβ-induced chemotaxis deficit in *Caenorhabditis elegans*

The chemotaxis behavior in *C. elegans* is regulated by stimulation of sensory neurons and inters neurons to activate the motor neurons (Hobert, 2003). To investigate whether EGCG, OFA and OFB could improve Aβ-induced deficit in chemotaxis behavior, worms of CL2355 strain were used (Figure 5D). OFA and OFB show better chemotaxis ability toward benzaldehyde compare with negative control. 50 μM EGCG, 5 μM OFA, and 5 μM OFB significantly increased chemotaxis index for 71.43% (*P* < 0.01), 68.25% (*P* < 0.05), and 58.77% (*P* < 0.05), respectively. Meanwhile, OFA and OFB didn’t affect the chemotaxis behavior of the control strain CL2122. These results suggest that OFA and OFB contain some protective effects against Aβ-induced chemotaxis deficit.

### Oolonghomobisflavan A and oolonghomobisflavan B extended the lifespan of *Caenorhabditis elegans*

In another set of experiments, we investigated whether OFA and OFB can increase the lifespan of *C. elegans* similarly as EGCG (Abbas and Wink, 2009). wild-type (N2) strain was used to detect the effect of OFA and OFB on lifespan (Supplementary Figure 2 and Supplementary Table 2). The mean lifespan of negative control was 14.92 ± 0.31 d, while OFA treated worms lived to 18.71 ± 0.40 d (5 μM), 19.55 ± 0.45 d (10 μM), and 19.83 ± 0.49 d (25 μM), respectively, OFB are 19.98 ± 0.58 (5 μM), 20.01 ± 0.46 (10 μM), and 18.50 ± 0.51 d (25 μM), respectively. These results show that OFA and OFB extend the lifespan of wild-type (N2) strain under normal condition.

### The antioxidant effects of oolonghomobisflavan A and oolonghomobisflavan B

OFA and OFB exhibit substantial antioxidant activity. When tested in the DPPH assay, both OFA and OFB effectively scavenged radicals (IC_50_ = 4.65 ± 0.26 μM and 4.80 ± 0.30 μM, respectively). These values are in a similar range as those of known antioxidant EGCG (Supplementary Table 3).

### Oolonghomobisflavan A and oolonghomobisflavan B increases the stress resistance in *Caenorhabditis elegans*

**FIGURE 6:**
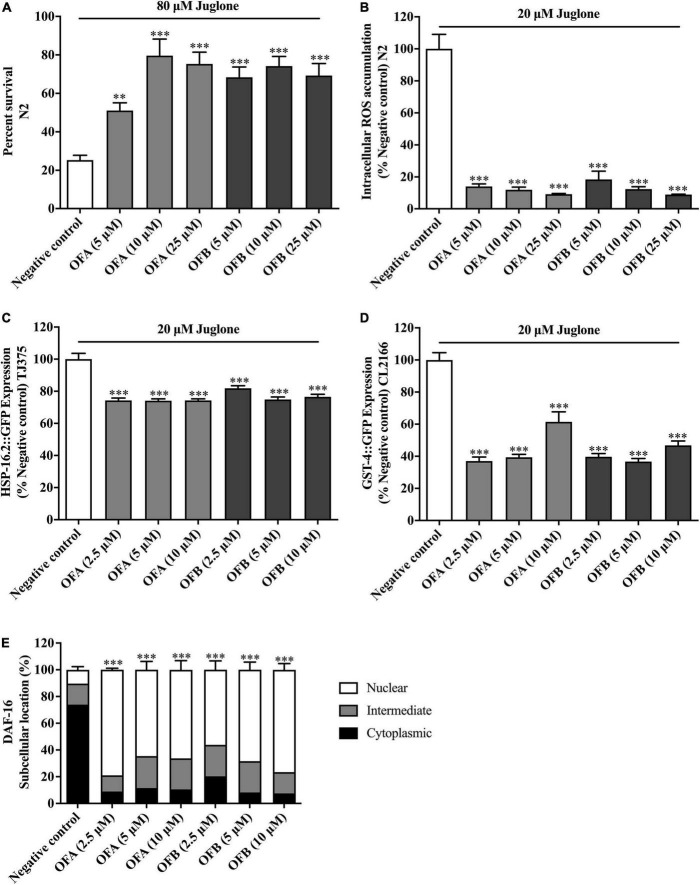
Effect of OFA and OFB on *C. elegans* under oxidative stress condition treated with juglone. Percent survival of wild-type (N2) treated with OFA and OFB under lethal juglone concentration (80 μM) condition **(A)**. OFA and OFB attenuate intracellular ROS accumulation in wild-type (N2) under 20 μM juglone condition **(B)**. Effects of OFA and OFB on the expression of *hsp-16.2* **(C)** and *gst-4* **(D)** under 20 μM juglone condition. The localization of DAF-16 transcription factors **(E)**. All data are shown as the mean ± standard deviation (*n* ≥ 3 independent experiments). ^**^p < 0.01, ^***^p < 0.001 compared to the negative control.

To further investigate the antioxidant properties of OFA and OFB, the survival of nematodes was analyzed under oxidative stress conditions. Wild-type (N2) strain was cultured in S-medium under oxidative stress which was induced by 80 μM juglone. We found that all concentrations of OFA and OFB significant increased the survival rate when compared with negative control. OFA (5, 10, and 25 μM) increased the survival rate to 25.79% (*P* < 0.05), 54.34% (*P* < 0.001), and 50.02% (*P* < 0.001), respectively, OFB (5, 10 and 25 μM) increased the survival to 43.11 (*P* < 0.001), 48.95% (*P* < 0.001), and 44.01% (*P* < 0.001), respectively compared with negative control (Figure 6A). These results show that OFA and OFB can increase the survival rate of wild-type (N2) under the lethal concentration of juglone.

### Oolonghomobisflavan A and oolonghomobisflavan B decreased the reactive oxygen species accumulation in *Caenorhabditis elegans*

To investigate whether EGCG, OFA, and OFB could suppress juglone-induced oxidative stress, the intracellular ROS accumulation assay was determined in wild-type (N2) worms. We found that OFA (5, 10, and 25 μM) significant decreased the ROS level 86.01, 88.12, and 90.87%, respectively compared with negative control, OFB (5, 10, and 25 μM) significant decreased the ROS level to 81.51, 87.71, and 91.13%, respectively compared with negative control (Figure 6B). These results suggest that OF can decrease the ROS accumulation in *C. elegans*.

### Oolonghomobisflavan A and oolonghomobisflavan B modulated the expression of stress response genes in *Caenorhabditis elegans*

To elucidate mechanism of action of OFA and OFB, the expression of the stress response related genes were examined in transgenic *C. elegans* (CL2166 and TJ375) (Figure 6). We found that OFA (2.5, 5, 10 μM) significantly decreased the expression of GST-4:GFP by 62.92, 60.59, and 38.53% compared with negative control (Figure 6D). OFB (2.5, 5, 10 μM) significantly decreased the expression of GST-4:GFP by 60.29, 63.29, and 53.18% compared with negative control (Figure 6D). OFA (2.5, 5, 10 μM) significantly decreased the expression of HSP-16.2:GFP by 25.71, 25.91, and 25.70% compared with negative control (Figure 6C). OFB (2.5, 5, 10 μM) significantly decreased the expression of HSP-16.2:GFP by 18.13, 25.12, and 23.43% compared with negative control (Figure 6C).

### Oolonghomobisflavan A and oolonghomobisflavan B increased the nuclear translocation of DAF-16 in *Caenorhabditis elegans*

DAF-16, a FOXO-family transcription factor, is the well-known regulator of aging in response to the IIS pathway in *C. elegans* (Murphy et al., 2003). The nuclear localization of DAF-16 is essential for activation stress resistance, and lifespan modulation genes. We next examined the effects of OFA and OFB on the nuclear localization of DAF-16. We found that OFA and OFB increased the nuclear translocation of DAF-16 (Figure 6E) in transgenic mutant *C. elegans*.

## Discussion

The accelerated aging of the population is now affecting the entire world. Neurodegenerative disorders have attracted considerable attention among the age-related disorders. Neurodegenerative diseases are linked to neuronal cell death and neurite outgrowth impairment that are often caused by oxidative stress. Plant bioactive secondary metabolites, which have antioxidative and neuroprotective properties could be potential candidates for alternative treatment of neurodegenerative diseases. This is the first report the beneficial effects of oolong tea polyphenols (OFA and OFB) promoting neuroprotective and neuritogenesis properties as well as anti-aging potential. Anti-aging in this study is concerned with extending the lifespan. Both OFA and OFB can extend the lifespan of *C. elegans* wild-type (N2) under normal cultured condition and increase the survival rate of wild-type (N2) under the lethal concentration of juglone.

Neurite outgrowth is a basal process of neuronal differentiation to promote neuronal regeneration after damage or nervous system disorder (Huang et al., 2016). In addition, neurite outgrowth is a crucial goal for the process of anti-neurodegenerative disease therapy (Hu et al., 2015). We found that OFA and OFB promoted neurite outgrowth and neurite-bearing cells in Neuro-2a cells. Besides morphological observation, neurite outgrowth marker examination was also an effective way for measuring neurite outgrowth. GAP-43 (growth-associated protein) (Korshunova and Mosevitsky, 2010) and Ten-4 (Teneurin-4) (Suzuki et al., 2014) are neuron-specific protein associated which regulates neurite outgrowth through axon growth and growth cone formation. We found that OFA and OFB increased GAP-43 and Ten-4 gene expression. Thus, OFA and OFB exhibited neuritogenesis properties by enhancing neurite outgrowth and neurite outgrowth proteins. The utilization of small molecules that interact with neurotrophic factors to stimulate neurite outgrowth at the location of nerve damage has been a potential therapeutic strategy (Bawari et al., 2019). At present, the neurotrophic factor family includes nerve growth factor (NGF), brain-derived neurotrophic factor (BDNF), neurotrophin-3 (NT-3), and neurotrophin-4 (NT-4). They mainly bind to the corresponding high-affinity tyrosine kinase receptors and initiate downstream signaling pathways to maintain neuron survival and promote nerve growth (Huang and Reichardt, 2003). Therefore, it is speculated that EGCG, OFA, and OFB may have physiological effects similar to neurotrophic factors.

The mechanism related to neuronal death in AD is still unclear, but excessive oxidative stress is regarded as major initiators or mediators of AD (Chen and Zhong, 2014). The aggregation of amyloid beta (Aβ) and neurofibrillary tangles enhanced the production of oxidative stress leading to neuronal cell death. In addition, excessive levels of glutamate can contribute to neuronal cell death through the mitochondrial function impairment and stimulation the ROS (Tobaben et al., 2011). We found that OFA and OFB exert a potent neuroprotective effects against cytotoxicity induced by excessive glutamate in cultured neuronal (Neuro-2a and HT22) cells by suppressing intracellular ROS production but also through enhancing the expression of endogenous antioxidant enzymes (*GSTo1*, *GSTa2*, *GPx*, *SOD1*, and *SOD2*).

Furthermore, we explored the neuroprotective effects *in vivo* by using transgenic worms which express human Aβ_1–42_ peptides. The CL4176 strain, which expresses Aβ_1–42_ peptides in muscle cells was used in paralysis assay. OFA and OFB delayed PT50 as compared to negative control. The CL2355 strain, which expresses Aβ_1–42_ peptides in neuron cells was used in chemotaxis assay. OFA and OFB improved chemotaxis ability toward benzaldehyde as compared with negative control worms. These results suggest that OFA and OFB exhibit protective effects against Aβ-induced toxicity in *C. elegans*. Taken together, OFA and OFB exhibit potent neuroprotective effects against glutamate/Aβ-induced toxicity both *in vitro* (Neuro-2a and HT22 cells) and *in vivo* (*C. elegans*). Numerous naturally occurring phytochemicals reduced the level of Aβ induced ROS level through its antioxidative property (Thapa and Carroll, 2017). Our results agree with the neuroprotective properties in tea polyphenol which have been reported in several studies (Abbas and Wink, 2009; Rho et al., 2019; Zhan et al., 2020).

Oxidative stress is considered as a major risk factor for age-related diseases (Wadhwa et al., 2018). In the last decade, the correlation between antioxidant and age-related diseases has gained a lot of attention. Some researchers announced a positive correlation between antioxidants in drinks and longevity (Sadowska-Bartosz and Bartosz, 2014). EGCG could increase lifespan in several animal models such as *Drosophila melanogaster* (Wagner et al., 2015), *Caenorhabditis elegans* (Abbas and Wink, 2009), and *Rattus norvegicus* (Niu et al., 2013). These studies are in agreement with our findings that OFA and OFB have longevity effect by extending the lifespan of *C. elegans* (Duangjan and Curran, 2022). Since OFA and OFB are dimers of EGCG, this result implies that even larger polyphenols exhibit bioavailability in worms. Interestingly, OFA and OFB also exhibit oxidative stress resistance properties in *C. elegans*. We found that OFA and OFB can effectively protect worms against severe oxidative stress and reduced the amount of intracellular ROS. The antioxidant effects of OFA and OFB were further confirmed by the *in vitro* DPPH assays. OFA and OFB exhibit powerful antioxidant activity in a similar range as EGCG. These results support the neuroprotective effects of OFA and OFB in cultured neuronal (Neuro-2a and HT22) cells which suppressed intracellular ROS and induced endogenous antioxidant enzymes. DAF-16, a FOXO-family transcription factor, is the well-known regulator of aging in response to the IIS pathway in *C. elegans* (Murphy et al., 2003; Guerrero-Rubio et al., 2020; Navarro-Hortal et al., 2021). DAF-16/FOXO remains inactive state in the cytoplasm until external factors such as stress or certain ligands motivate DAF-16/FOXO translocation from the cytoplasm to the nucleus, inducing the expression of stress response gene including *sod-3*, *gst-4*, and *hsp-16.2* (Peixoto et al., 2016; Wang and Wink, 2016; Duangjan et al., 2019b; Li et al., 2019). We found that OFA and OFB increased the nuclear translocation of DAF-16. Moreover, OFA and OFB modulated the expression of stress response genes by down-regulation of *hsp-16.2* and *gst-4* gene expression under juglone induced-oxidative in *C. elegans*. Potential antioxidant activities of tea polyphenols such as EGCG, catechin and caffeine in *C. elegans* were also reported from previous studies (Abbas and Wink, 2009, 2014; Li et al., 2019). Tea has been shown a potential therapeutic agent for preventing age-related diseases such as cancer, cardiovascular diseases and neurodegenerative diseases. This ability is mostly attributed to their antioxidant activity of polyphenols, including the major component EGCG. However, we found that OFA and OFB exhibited the higher neuroprotective than EGCG. The enhanced anti-oxidant activity, is possibly due to existence of more hydroxyl groups, and cytoprotective effects of bi-flavonoids compared to flavonoid monomers (Shin et al., 2006; Hamaguchi et al., 2009; Thapa and Chi, 2015). Dimers of apigenin revealed enhanced anti-cancer effects compared to apigenin (flavonoid monomer) alone (Chan et al., 2006; Hadden and Blagg, 2008). Both OFA and OFB contain a methylene bridge linking the 8,8’-positons of two flavan units. Maybe it is the key why OFA and OFB showed a better neuroprotective effect than EGCG. Taken together, this study provides the first evidence of OFA and OFB in neuritogenesis and neuroprotective potential both *in vitro* (Neuro-2a and HT22 neuronal cells model) and *in vivo* (*C. elegans* model). OFA and OFB protected Neuro-2a and HT22 neuronal cells against glutamate-induced toxicity and of *C. elegans* against juglone/Aβ-induced toxicity. OFA and OFB significantly reduced the intracellular ROS accumulation and regulated the gene expression of antioxidant enzymes in these models. According to an examination of 302 neurons, the neurotransmitters, vesicle circulation and synaptic transmission of the nematode are highly conserved. Thus, *C. elegans* has been used as an *in vivo* model for neurotoxicity (Link et al., 2016). Amyloid-β, a well-known hallmark of AD, can induce oxidative stress and neuronal cell death, which plays an important role in AD (Yankner and Lu, 2009). Moreover, we got the positive results from paralysis assay (CL4176 model, which expresses Aβ_1–42_ peptides in muscle cells) and chemotaxis assay (CL2355 model, which expresses Aβ_1–42_ peptides in neuron cells) treated with OFA and OFB, which proved the neuroprotective effect against Aβ-induced toxicity. Considering the AD is age-related neurodegenerative disease, we deduced that OFA and OFB may contain the anti-aging effect. And it’s consistent with our findings that OFA and OFB significantly extend the lifespan of wild type (N2) *C. elegans*. The oolong tea polyphenols (OFA and OFB) possesses both longevity promoting effects and neuroprotective activity supporting its therapeutic potential for the treatment of age-associated neurodegenerative diseases. Further studies are required to study the molecular mechanism of OFA and OFB on longevity and neuroprotective properties, and *in vivo* tests with more complex model organisms and intervention studies.

## Conclusion

In conclusion, OFA and OFB increased the neurite length of Neuro-2a, decreased the accumulation of ROS in cultured neuronal cells (Neuro-2a and HT22), up-regulated the expression of antioxidant mRNA expression (*GPx*, *GSTs*, and *SODs*). Moreover, OFA and OFB delayed the Aβ-induced paralysis in transgenic strain CL4176 and counteracted the chemotaxis deficit in strain CL2355. Similar with EGCG, OFA, and OFB can extend the lifespan of *C. elegans*. OFA and OFB maybe become promising candidates agents for treating the age-related neuroprotective diseases in the future.

## Data availability statement

The original contributions presented in this study are included in the article/Supplementary material, further inquiries can be directed to the corresponding author/s.

## Authors contributions

SZ and CD performed the experiments, analyzed the data, and were major contributors in writing the manuscript. SZ, CD, TT, and LW designed the study and prepared media and reagents. JL and MW provided materials for the study, conceived and supervised the research, and reviewed and edited the manuscript. JL, MW, TT, and LW corrected the manuscript. All authors contributed to manuscript revision, read, and approved the submitted version.

## Abbreviations

EGCG: Epigallocatechin gallate
OFA: Oolonghomobisflavan A
OFB: Oolonghomobisflavan B
MTT: 3-(4,5-Dimethylthiazol-2-yl)-2,5-diphenyltetrazolium bromide
FBS: Fetal bovine serum
DMSO: Dimethyl sulfoxide
LDH: Lactate dehydrogenase
A β: β -amyloid
H2DCFDA: 2′, 7′ -Dichlorodihydrofluorescein diacetate
AD: Alzheimer’s disease
PT_50_: Paralyzed time in 50%
ROS: Reactive oxygen species.
