# Supplementary material for: Oolonghomobisflavans exert neuroprotective activities in cultured neuronal cells and anti-aging effects in Caenorhabditis elegans
Source: Front Aging Neurosci. 2022 Sep 7;14:967316. doi: 10.3389/fnagi.2022.967316 (PMC9490402; doi:10.3389/fnagi.2022.967316)
Supplement: Supplementary file 1 [file Data_Sheet_1.DOCX]

**Supplementary Material**


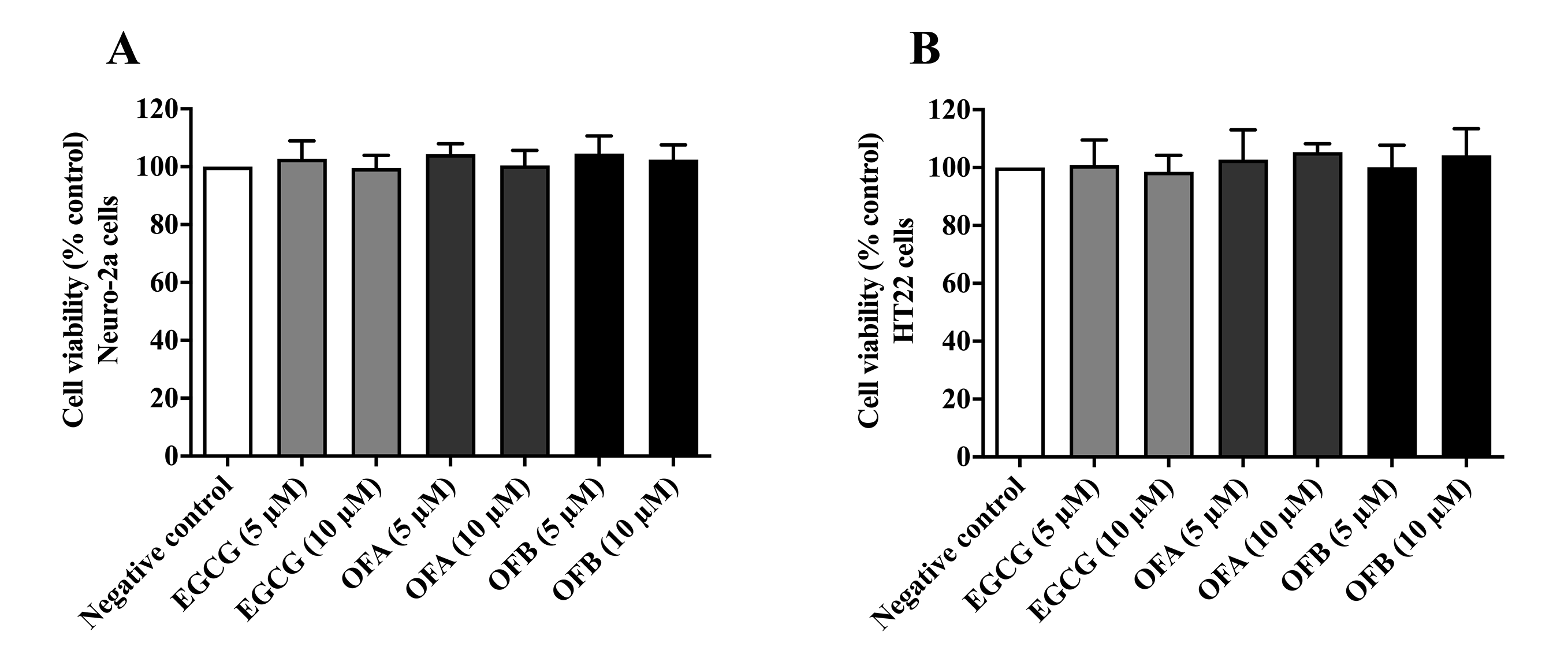


**Figure S1** Effect of EGCG, OFA and OFB on cell viability in neuronal (Neuro-2a and HT22) cells. Cells were treated with different concentrations of EGCG, OFA and OFB for 1 h. (A) Cell viability of Neuro-2a. (B) Cell viability of HT22. All data are shown as the mean ± standard deviation (n ≥ 3 independent experiments); *p* ≤ 0.001 compared to the negative control.


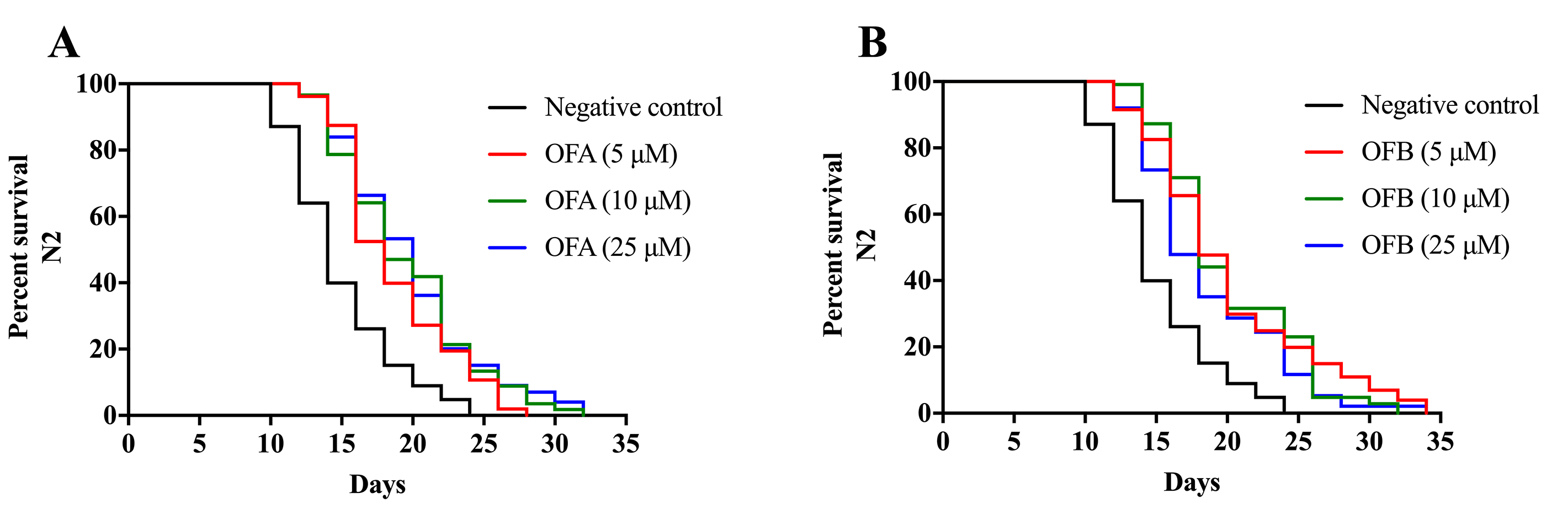


**Figure S2** OFA (A) and OFB (B) extend the lifespan of *C. elegans*. The data were performed by Kaplan-Meier in software SPSS24, survival curves were drawn by GraphPad Prism 8 software.

**Table S1** PT_50_ values of CL4176 treated with OFA and OFB

| Treatment | PT_50_ ± SEM (h) | *P* value | Significance |
| --- | --- | --- | --- |
| Negative control | 22.90 ± 0.15 |  |  |
| EGCG (50 μM) | 24.74 ± 0.24 | 0.0010 | ** |
| OFA (5 μM) | 24.65 ± 0.43 | 0.0037 | ** |
| OFA (10 μM) | 25.06 ± 0.25 | 0.0007 | *** |
| OFA (25 μM) | 24.83 ± 0.31 | 0.0018 | ** |
| OFB (5 μM) | 25.91 ± 0.26 | 0.0004 | *** |
| OFB (10 μM) | 25.01 ± 0.42 | 0.0003 | *** |
| OFB (25 μM) | 24.58 ± 0.04 | 0.0017 | ** |

Note: PT_50_ means time required for 50% of the worms to show paralysis; ***p*<0.01, ****p* < 0.001 compared with the negative control.

**Table S2** The effects of OFA and OFB on the lifespan of *C. elegans*

| Strains | Treatment  (μM) | Mean lifespan  (mean ± SEM) | Log rank  *P* value |
| --- | --- | --- | --- |
| wild-type (N2) | Control | 14.92 ± 0.31 |  |
|  | OFA 5 | 18.71 ± 0.40 | < 0.001 |
|  | OFA 10 | 19.55 ± 0.45 | < 0.001 |
|  | OFA 25 | 19.83 ± 0.49 | < 0.001 |
|  | OFB 5 | 19.98 ± 0.58 | < 0.001 |
|  | OFB 10 | 20.01 ± 0.46 | < 0.001 |
|  | OFB 25 | 18.50 ± 0.51 | < 0.001 |

Note: The mean lifespan was calculated by Kaplan-Meier in software SPSS24; the comparison between the two groups of survival curves was calculated by Log Rank (Mantel-Cox) test.

**Table S3** *In vitro* evaluation of antioxidant effect

| Compounds | EGCG | OFA | OFB |
| --- | --- | --- | --- |
| IC_50_ (µM) | 7.51 ± 0.49 | 4.65 ± 0.26 | 4.80 ± 0.30 |

Note: Results were represented as mean ± standard deviation.
